# Bio-distribution and toxicity potential of human umbilical cord mesenchymal stem cells in cynomolgus monkeys

**DOI:** 10.1038/s41598-024-63118-4

**Published:** 2024-05-28

**Authors:** Ying Huang, Xiaofang Hao, Zhi Lin, Lulu Li, Hua Jiang, Hezhan Zhang, Xingchao Geng, Hao Zhu, Hairuo Wen

**Affiliations:** 1 Key Laboratory of Beijing for Nonclinical Safety Evaluation Research of Drugs, National Center for Safety Evaluation of Drugs, National Institutes for Food and Drug Control, A8 Hongda Middle Road, Beijing, Economic-Technological Development Area, Beijing, 100176 People’s Republic of China; 2 Sinoneural Cell Engineering Group Co., Ltd., Shanghai, People’s Republic of China

**Keywords:** Cell therapy, Human embryonic stem cell differentiation-derived mesenchymal stem cells, Cynomolgus monkey, Toxicity, Safety, Bio-distribution, Mesenchymal stem cells, Toxicology

## Abstract

Mesenchymal stem cells (MSCs) have demonstrated promising advantages in the therapies of many diseases, while its multi-directional differentiation potential and immunotoxicity are the major concerns hindered their clinical translation. In this study, human umbilical Mesenchymal stem cell (hUC-MSCs) were labeled with a near-infrared fluorescent dye DiR before infused into cynomolgus monkeys, and the amount of hUC-MSCs in the peripheral blood were dynamically estimated from 5 min to 28 days post a single administration at 3 × 10^6^ cells/kg and 2 × 10^7^ cells/kg intravenously. As results, some hUC-MSCs distributed to the whole body within 5 min, while most of the cells accumulate in the lungs along with the systemic blood circulation, and subsequently released into the blood. The toxicity potentials of hUC-MSCs were investigated in another 30 cynomolgus monkeys, and the cells were repeatedly administrated at doses of 3 × 10^6 ^cells/kg and 2 × 10^7^ cells/kg for 5 times on a weekly basis, with a recovery period of 1 months. hUC-MSCs showed no obvious toxic effects in cynomolgus monkeys, except xenogeneic immune rejection to human stem cells. Low levels of the hUC-MSC gene were detected in the peripheral blood of a few animals administered 2 × 10^7^ cells/kg at 30 min subsequent to the first and last administration, and there was no significant difference in the copy number of the hUC-MSC gene in the blood samples compared with the first and last administration, indicating that the hUC-MSC was not significantly amplified in vivo, and it its safe in non-human primates. Our study for the first time verified the safety of long-term use of hUC-MSCs in primates. We have pioneered a technology for the real-time detection of hUC-MSCs in peripheral blood and provide dynamicand rapid monitoring of the distribution characteristics of hUC-MSCs in vivo. Here, we provide data supporting the application of such products for clinical treatment and the application of stem cells in major refractory diseases and regenerative medicine.

## Introduction

Stem cell therapy is a novel treatment, in which human autologous or allogeneic stem cells are transplanted into the human body subsequent to in vitro manipulation, to repair diseased cells or rebuild normal cells and tissues^1^. It has demonstrated advantages in immune diseases, metabolic diseases, cardiac diseases, ophthalmic diseases and skin diseases, and is considered as one of the most cutting-edge and promising fields in medical research today^2,3^. For instance, mesenchymal stem cells (MSCs) with benefits in multidirectional differentiation potential, hematopoietic supply, promotion ofstem cell engraftment, immune regulation and self-replication, has attracted increasing attention^4,5^.

Till now, there are over 8000 clinical trial conducted and 14 stem cell-derived cell therapy products have been approved for new drug application (NDA) internationally. However, as a special new biotherapeutic product, stem cells have demonstrated a series of risks, such as tumorigenicity, tumor promotion, and immunogenicity. Previous studies have shown that ESCs and induced pluripotent stem cells (iPSCs) could induce benign or malignant teratomas, and high-passage murine MSCs could spontaneously transform into malignant tumors. Stem cells may also affect the growth and proliferation of existing tumor cells in the body, a characteristic known as the "tumorigenicity" risk of stem cells^6^. The clinical application of stem cells may also face biological risks come from their immunogenic and immunomodulatory properties. The immunogenicity of MSCs before differentiation is low, but they could be highly immunogenic after differentiation due to the expression of major histocompatibility complex (MHC) molecules^7^. In addition, the in vivo homing and differentiation mechanism of stem cells remain unclear, and there may be risks of non-targeted site transfer and differentiation^8^. Therefore, a method to predict the safety of stem cells in the human body by preclinical evaluation in animals is an urgent need^9–13^. Since each stem cell product has unique characteristics and theraputic methods, and its bio-distribution, tumorigenicity and other specific safety risks differ from those of other biological products, it is difficult to identify a single fixed preclinical evaluation model. With the continuous research and development of a large number of innovative stem cell therapy products, it is becoming urgently necessary to solve the obstacles in the evaluation of the safety of mesenchymal stem cells by obtaining nonclinical safety-related data and information as much as possible to promote the development and clinical application of stem cells.

Human umbilical cordmesenchymal stem cells (hUC-MSCs) are intended to treat multiple immune diseases (for instance, psoriasis, liver failure, and lupus nephritis) by intravenously infusion^14,15^. Compared with human bone marrow and adipose derived mesenchymal stem cells, they are easier to obtain, have demonstrated strong proliferation and differentiation capability and strong plasticity, with less ethical controversy and has less cell loss after cryopreservation. In recent years, many clinical studies have shown that stem cell could promote endometrial growth and reduce endometrial fibrosis, thereby improving the uterine cavity environmental unique advantage^16–21^. Despite the favorable preliminary research results of stem cells in the treatment of endometrial aplastic diseases, many concerns still stood out: for instance, the survival and distribution of cells in vivo following transplantation, abnormal proliferation in vivo, off-target toxic reactions, and abnormal immune responses, among others. In light of this, preclinical exploration and verification studies based on animal models are indispensable.

In this study, we administered DIR-labeled hESC-MSCs to cynomolgus monkeys once to study the in vivo bio-distribution characteristics in peripheral blood over 4 weeks. To fully investigate the toxicity risk of the cells, two different doses of hUC-MSCs were administered intravenously to cynomolgus monkey for 5 times with a week interval, and recovered for 4 weeks. To understand the distribution characteristics, the content of hUC-MSCs were determined by q-PCR by the end of dosing period and recovier period, respectively. We provide important data supporting the clinical application of stem cells.We have pioneered a technology for the real-time detection of hUC-MSCs in peripheral blood and provide dynamic and rapid monitoring of the distribution characteristics of hUC-MSCs in vivo. We also verified the safety of long-term use of hUC-MSCs in primates for the first time. Here, we provide data supporting the application of such products for clinical treatment and the application of stem cells in major refractory diseases and regenerative medicine. hUC-MSCs used in this study has been approved for clinical trial in China for the treatments of active inflammatory bowel disease, liver failure, severe lupus nephritis, and chronic plaque psoriasis.

## Materials and methods

### Cells

hUC-MSCs at GMP-grade quality were provided by Sinoneural Cell Engineering Group Co., Ltd. and stored in liquid nitrogen away from light. The stem cells were recovered and using solvent (saline containing 5% DMSO and 5% human blood albumin, the DMSO was further diluted and the final concentration in the cell suspension was 0.25%), and were adjusted to a certain concentration before use, and the viability was estimated as 89.00% ± 3.43%.

### Animals

A total 34 of cynomolgus monkey (*Macaca fascicularis*) aged 3–4 years were purchased from Guangdong Blue Island Biotechnology Co., Ltd. (Experimental animal production license number: SCXK [Yue] 2019–0010). The the body masses of females ranged from 2.50 to 3.25 kg, and the body masses of males ranged from 2.55 to 3.80 kg. The temperature of the rearing room was 16–26 °C, the relative humidity was 40–70%, and the lighting time was 12 h/day. All animal experimental procedures were approved by the Institutional Animal Care and Use Committee (IACUC) of the National Center for Safety Evaluation of Drugs (No. IACUC-2020–069). The study is presented in accordance with Animal Research: Reporting of In Vivo Experiments (ARRIVE) guidelines.

### Groups and administration

There are four animals used in the bio-distribution study with DiR, and 30 animals in the toxicity study. All the cynomolgus monkey for the toxicity study were randomlised into 3 groups, including Control Group, Low-dose Group and High-dose Group, with 5 males and 5 females in each. These animals were administered intravenously (intravenous infusion at 3 mL/min) with solvent or hUC-MSCs at 3 × 10^6^ cells/kg or 2 × 10^7^ cells/kg at 10 mL/kg for once a week for 4 consecutive weeks (five times in total), and a 4-week recovery period was set after the last administration.

The intended clinical dose of hUC-MSCs is 1 × 10^6^ cells/kg. In terms of kilogram body weight, the low dose (3 × 10^6^ cells/kg) and high dose (2 × 10^7^ cells/kg) used in this study was 3 times and 20 times the clinical equivalent dose, respectively.

### Bio-distribution of hUC-MSCs with DiR

hUC-MSCs were rinsed with PBS for twice before use. Dye of 1, 1′-dioctadecyl-3,3,3′,3′-tetramethylindotricarbocyanine iodide (DiR, Caliper Life Sciences) was diluted to 5 μg/mL andmixed with the hUC-MSCs (for 1 × 10^6^ cells, 1 mL of DiR dye was required). The mixture was incubated in a 37 °C incubator for 30 min, and the supernatant was removed by centrifugation at 400 g for 5 min. The cells were rinsed again in PBS for twice by centrifugation. Four monkeys were used in this study. DiR-labeled hUC-MSCs were administered to monkeys by a single intravenous injection at doses of 3 × 10^6^ cells/kg or 2 × 10^7^ cells/kg respectively. Peripheral blood of 500 μL was collected at before, 0.083 h, 0.25 h, 0.5 h, 1 h, 2 h and 3 h, 1, 2, 3, 5, 7, 10, 14, 21, and 28 days post-administration to determine the distribution of hUC-MSCs in blood using FACS Calibur flow cytometry (Becton, Dickinson and Company, U.S.A). In the single administration study, hUC-MSCs were labeled with near-infrared dye DIR, and the metabolic characteristics of hESC-MSCs in the blood of cynomolgus monkeys were continuously and dynamically detected by flow cytometry.

### Repeated dosed toxicity of hUC-MSCs

**Figure 1:**
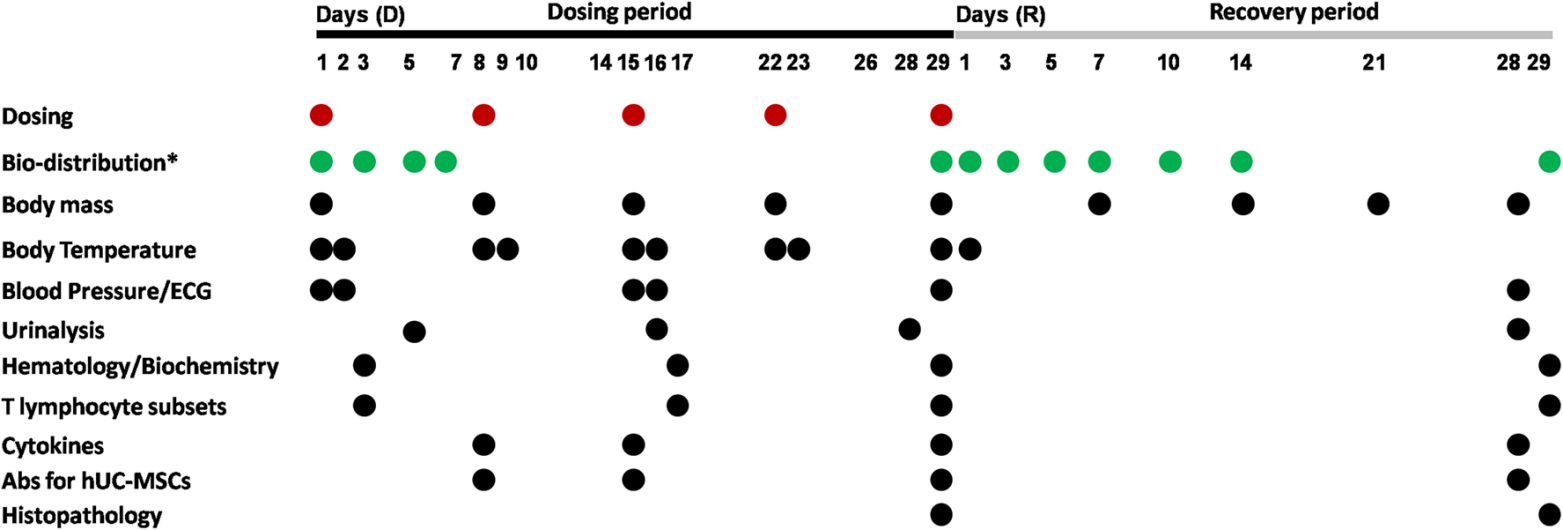
Study schedule of toxicity study. hUC-MSCs were repeated administered to monkeys on D1, D8, D15, D22, and D29, respectively. *For bio-distribution, blood samples were collected on D1 (at before, 0.083 h, 0.5 h, 1 h, and 3 h), D3, D5, and D7, and D19 (at 0, 0.083 h, 0.5 h, 1 h, and 3 h), R1, R3, R5, R7, R10, R14, and R29. Clinical symptoms were observed every day, a body weight were measured every week. Body temperature was determined on the day and the following day of administration. ECG and blood pressure were measured on D1, D2, D15, D16, D28, R28. Urinalysis was performed on D5, D16, D28, and R28.Blood samples were collected on D3, D17, D29, and R29 for hematology, biochemistry, T lymphocyte subsets detection, respectively. Antibodies for hUC-MSCs were detected on D8, D15, D29, and R29. Animals were anesthetized on D29 and R29 for gross and histopathological examination.

**Figure 2:**
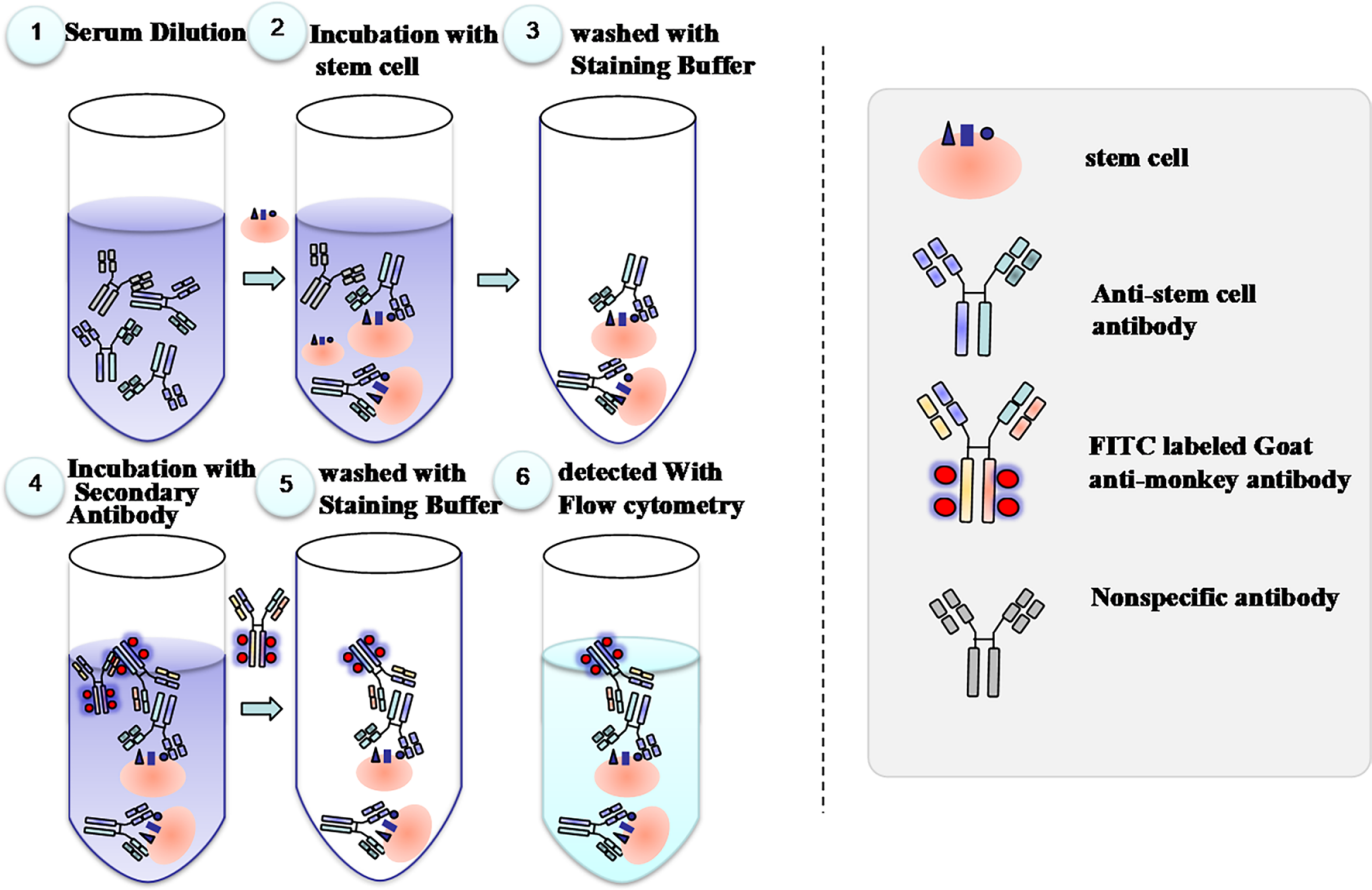
The Flow cytometry test for anti-stem cell antibodies. Animal serum samples were collected at different time points, diluted and incubated with stem cells. Subsequent to the addition of labeled secondary antibodies, flow cytometry was used to detect the presence of anti-stem cell antibodies in the samples.

See Fig. 1 for detailed study schedule. Clinical symptoms were observed daily, observing the appearance, coat, activity, neurological signs, respiratory status, and body posture.All injection sites were examined for redness, swelling, induration, suppuration and necrosis. Body mass was measured with a floor scale. Body temperature was measured by anal thermometer. The electrocardiogram (ECG) was recorded, and the indicators were calculated on the basis of the ECG recording, including heart rate, PR intervals, RR intervals, QRS durations, QT intervals, corrected QT intervals (QTc). Blood pressure was determined by non-invasive sphygmomanometry, including systolic blood pressure (SBP), diastolic blood pressure (DBP), and mean blood pressure (MBP). Urinalysis (including glucose, protein, bilirubin, urobilinogen, pH, specific gravity, occult blood, ketones, nitrite, white cell, and color) were performed using an AUTION AE-4020 automated urine chemistry analyzer. Blood was collected from a forearm vein. Hematologic analysis was performed using an ADVIA 2120 analyzer (Siemens; Munich, Germany), including white blood cell count (WBC), neutrophil count (Neut), lymphocyte count (Lymph), monocytecount (Mono), eosinophil count (Eos), basophil count (Baso). Serum was isolated and used for biochemical analysis using the automatic biochemical analyzer (7180, Hitachi, Japan), including alanine aminotransaminase (ALT), spartate aminotransaminase (AST), alkaline phosphatase (ALP), creatine kinase (CK), gamma glutamyl transpeptidase (GGT), lactate dehydrogenase (LDH), total bilirubin (TBIL), urea nitrogen (UREA), creatinine (CRE), glucose (GLU), total cholesterol (CHO), triglyceride (TG), total protein (TP), albumin (ALB), albumin/globulin ratio (A/G), serum potassium (Na^+^), serum natrium (K^+^), serum chlorine (Cl^-^), IgA, IgG, IgM, C3, and C4. Immunophenotyping of T lymphocyte was assessed by flow cytometry, including CD3^+^/CD4^+^T lymphocyte, CD3^+^/CD8^+^T lymphocyte, CD4^+^/CD8^+^ ratio, CD4^+^CD25^+^Foxp3^+^ regulatory T cells (Treg). Flow cytometry was establishedto test the immunogenicity of stem cells (Fig. 2). hUC-MSCs were incubated with diluted serum samples for 30 min before incubating with goat anti rhesus IgG (H + L) antibody for the detection of anti-hUC-MSCs binding antibodies. The levels of TNF-α, IFN-γ, IL-2, IL-4, IL-5, and IL-6 in monkeys serum were evaluated by a BD Human Th1/Th2 Cytokine Kit II (BD, U.S.). The monkeys were anesthetized with an intramuscular injection of Zoletil 50 (0.1 mL/kg). Gross and histopathological examinations were performed, including: brain, pituitary gland, thyroid gland (with parathyroid glands), submandibular gland, spinal cord (cervical, thoracic, lumbar segment), thymus gland, sternum (bone marrow), heart, aorta, tongue, trachea, oesophagus, lungs (with bronchus), liver, gallbladder, kidneys, adrenal glands, spleen, pancreas, stomach, duodenum, jejunum, ileum, cecum, colon, rectum, testes, epididymis, prostate gland, seminal vesicles, ovaries, fallopian tubes, uterus (with cervix), vagina, bladder, bone (unilateral thigh bone), sciatic nerve, muscle (skeletal muscle), skin, mammary glands (females only), eyeballs, optic nerves, mesenteric lymph nodes, inguinal lymph nodes, site of injection, and site of lesion. Tissues and organs weighed include: brain, thyroid (including parathyroid), thymus, heart, lungs (including bronchi), liver, kidneys, adrenal glands, spleen, testes, epididymis, ovaries, uterus (with cervix).

In addition, peripheral blood of 300 μL was collected at before, 0.083 h, 0.5 h, 1 h, 3 h, 1, 3, 5, and 7 days post the first administration, as well as before, 0.083 h, 0.5 h, 1 h, 3 h, 1, 3, 5, 7, 10, 14, and 29 days post the final administration. Tissue samples of blood, heart, liver, spleen, lungs, kidneys, brain, testes, epididymides, uterus, ovaries, stomach, intestine, fat, and skeletal muscle were collected, and the distribution of hUC-MSCs in blood was detected by Q-PCR method. DNA is extracted from various tissues using a blood and tissue kit (Qiagen, U.S.) and the concentration of elution DNA is adjusted to the actual range. The upstream and downstream primer sequences are "TGTCTTTTTCTTGTTTTGGAGGAA" and "CCAGACCACCCATAATCTTGTGT", respectively, and the probe sequences are "AGGAGCCCATCGGG". The primers above were designed and synthesized by Shanghai Shenggong Biological Engineering Co., Ltd. PCR reaction conditions were: 95 °C for 10 min, followed by 40 cycles of 95 °C for 15 s and 60 °C for 60 s. In this study, we used a TaqMan assay, in which a pair of primers and a specific fluorescent oligonucleotide probe was used. For each sample, three replicates were analyzed and the average of these replicates was calculated. Prior to PCR assay, DNA concentrations were measured with NanoDrop One to obtain genomic DNA content, and these concentrations were used to calculate the number of gene copies per nanogram of genomic DNA.

Animals were anesthesia by 35 mg/kg pentobarbital sodium, blood collection and necropsy were performed in a specific order (the Control Group, followed by the Low-dose Group and High-dose Group) to minimize potential confounders.

### Statistical analysis

All data are presented as the mean ± standard deviation (SD) of *n* values, where* n* corresponds to the number of mice used. Statistical analyses were performed using one-way ANOVA, followed by Dunnett’s test for comparisons to the Control group. Figures were generated using GraphPad Prism 5 for Windows (GraphPad Software, San Diego, CA, USA). Statistical significance was determined using SPSS (ver.26), with *p* < 0.05 considered significantly different.

### Ethical statement

The authors are accountable for all aspects of the work in ensuring that questions related to the accuracy or integrity of any part of the work are appropriately investigated and resolved. All animal experimental procedures were approved by the Institutional Animal Care and Use Committee (IACUC) of the National Center for Safety Evaluation of Drugs (NCSED, IACUC approval No. IACUC-2018–K001), in compliance with the IACUC Constitution of NCSED, the Guide for the Care and Use of Laboratory Animals (https://grants.nih.gov/grants/olaw/Guide-for-the-Care-and-use-of-laboratory-animals.pdf) and AAALAC International’s Position Statement. All study protocols (including the research question, key design features, and analysis plan) were prepared before the study and archived at National Center for Safety Evaluation of Drugs.

## Results

### Bio-distribution of hUC-MSCs

**Figure 3:**
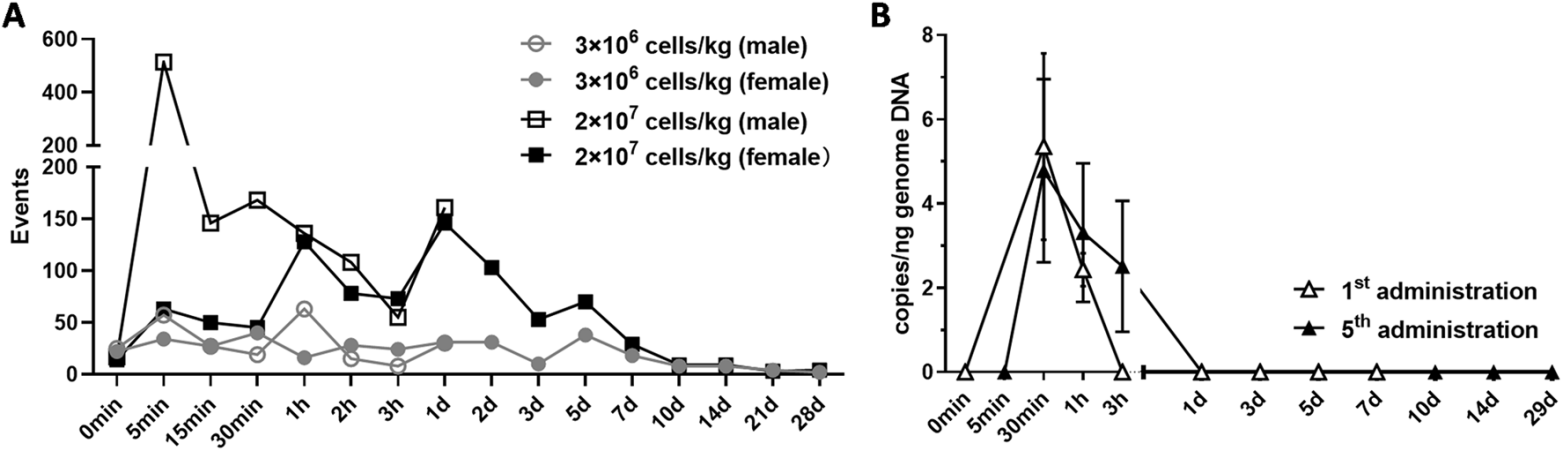
Bio-distribution in the peripheral blood of monkeys administered hUC-MSCs. (**A**) hUC-MSCs were stained with DiR before administered to the monkeys (n = 2/group), and the DiR^+^hUC-MSCs in peripheral blood were detected at before, 0.083 h, 0.25 h, 0.5 h, 1 h, 2 h, 3 h, 1 d, 2 d, 3 d, 5 d, 7 d, 10 d, 14 d, 21 d, and 28 d, respectively. (**B**) The DNA level of hUC-MSCs were measured at different time points after the first (1st) administration and final (5th) administration of 2 × 10^7^ cells/kg hUC-MSCs in the peripheral blood of monkeys (n = 10/group).

Bio-distribution data is a crucial piece of evidence in elucidating the safety of hUC-MSCs for clinical use. To investigate the distribution characteristics after re-infusion, a method combining in vitro labeling with near-infrared dye DIR and in vivo flow cytometry detection was established. It presents the advantages of simplicity, real-time, and rapid in the dynamic monitoring of stem cells in peripheral blood. Taking the percentage of hUC-MSCs in the blood as the detection index, the amount of hUC-MSCs reached a small peak at 5 min after administration, then gradually decreased, and began to rise at 1 h, reached the peak on 1 d after administration, and gradually decreased afterwards (Fig. 3).

In the toxicity study, the gene copy of hUC-MSCs could only be detected in the peripheral blood of animals in High-dose group at 30 min and 1 h post the initial administration, and reached the peak value at 0.5 h (5.35 copies/ng ± 2.21 copies/ng); after the final administration, the gene copeis of hUC-MSCs were only be detected in the peripheral blood of animals in High-dose group at 0.5 h, 1 h and 3 h post-administration, and reached the peak value at 0.5 h (4.78 copies/ng ± 2.17 copies/ng). See Fig. 3 for details. The gene copies of hUC-MSCs were all below the lower limit of quantification in all other tissues in all the animals.

### General toxicity

During the study period, no abnormal symptom was observed in all the animals in the solvent control group and the low dose group. Facture on the left upper limb was observed in a high dose female animal (FM303) from 8 to 23 days post the initial administration. Redness at the injection site was observed in all the groups and the incidence was 80% in solvent control group, 80% in low dose group and 90% in high dose group, and all the animals were recovered afterwards.

**Figure 4:**
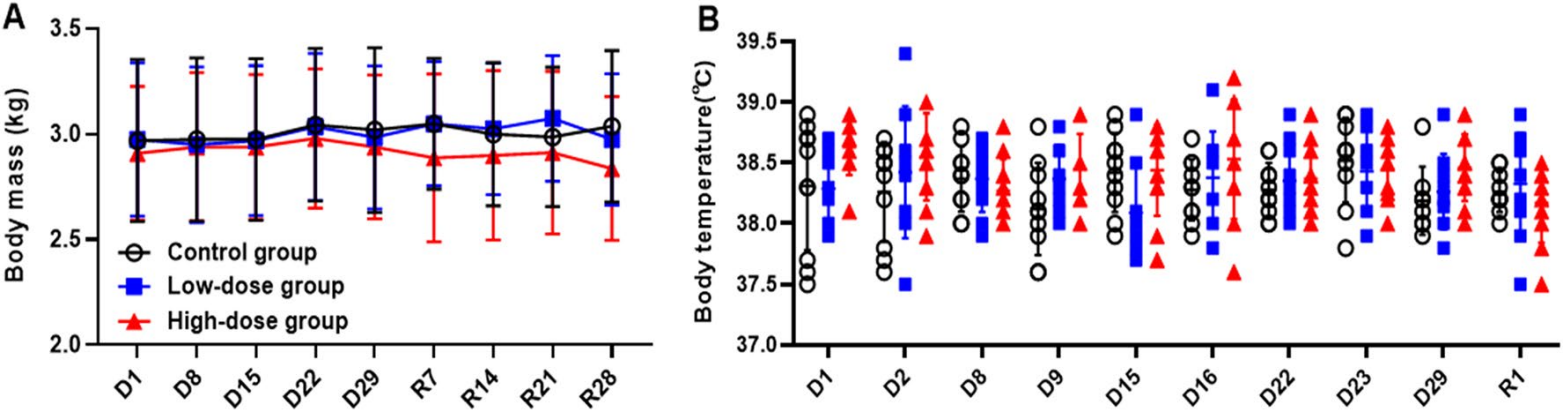
Changes on the body mass and temperature in monkey administered hUC-MSCs. Changes on the body mass (**A**) and body temperature (**B**) of monkeys during the study. (n = 10/group during dosing period; n = 4/group during recovery period).

**Figure 5:**
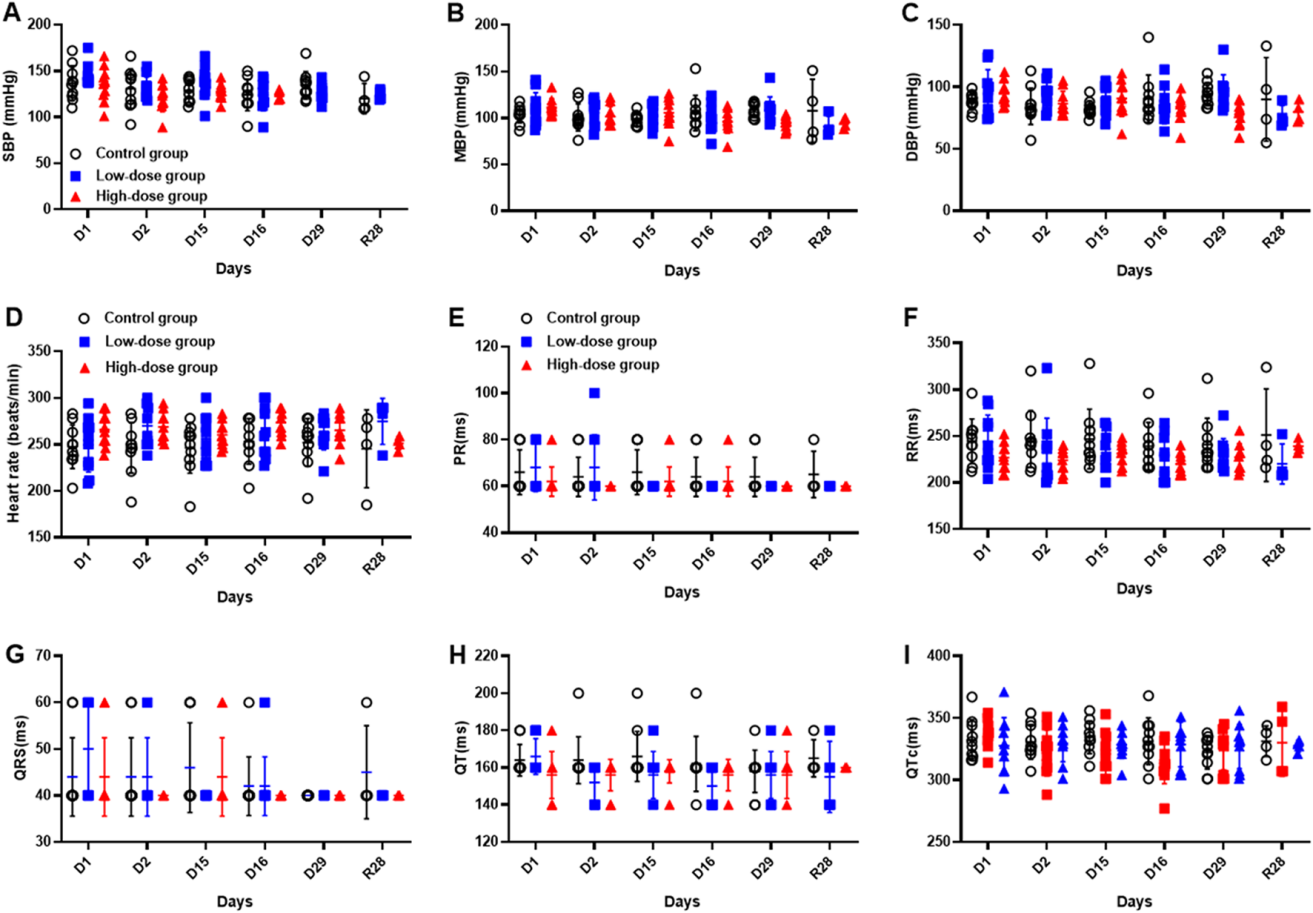
Changes on the blood pressure, heart rate and ECG indexes in monkey administered hUC-MSCs. Body pressure, including SBP (**A**), DBP (**B**), and MBP (**C**), as well as ECG indexes, including heart rate (**D**), PR (**E**), RR (**F**),QRS (**G**), QT (**H**), and QTc (**I**) are shown. (n = 10/group during dosing period; n = 4/group during recovery period).

Compared with the Control Group, no difference was found in averaged body masses of Low-dose group and High-dose group during the study period (Fig. 4). It is considered that the infusing of hUC-MSCs had no effect on the body mass of monkeys. During the study period, the body temperature of animals basically fluctuated within the normal range (around 37.5–39.5 °C for cynomolgus monkeys), and no difference was found comparing with the Control Group (Fig. 4). Blood pressure and electrocardiogram values of all the cynomolgus monkeys were also within the normal range, as no obvious difference associated with the administration of hUC-MSCs were observed (Fig. 5).

### Hematology, serum biochemistry and urinalysis

**Figure 6:**
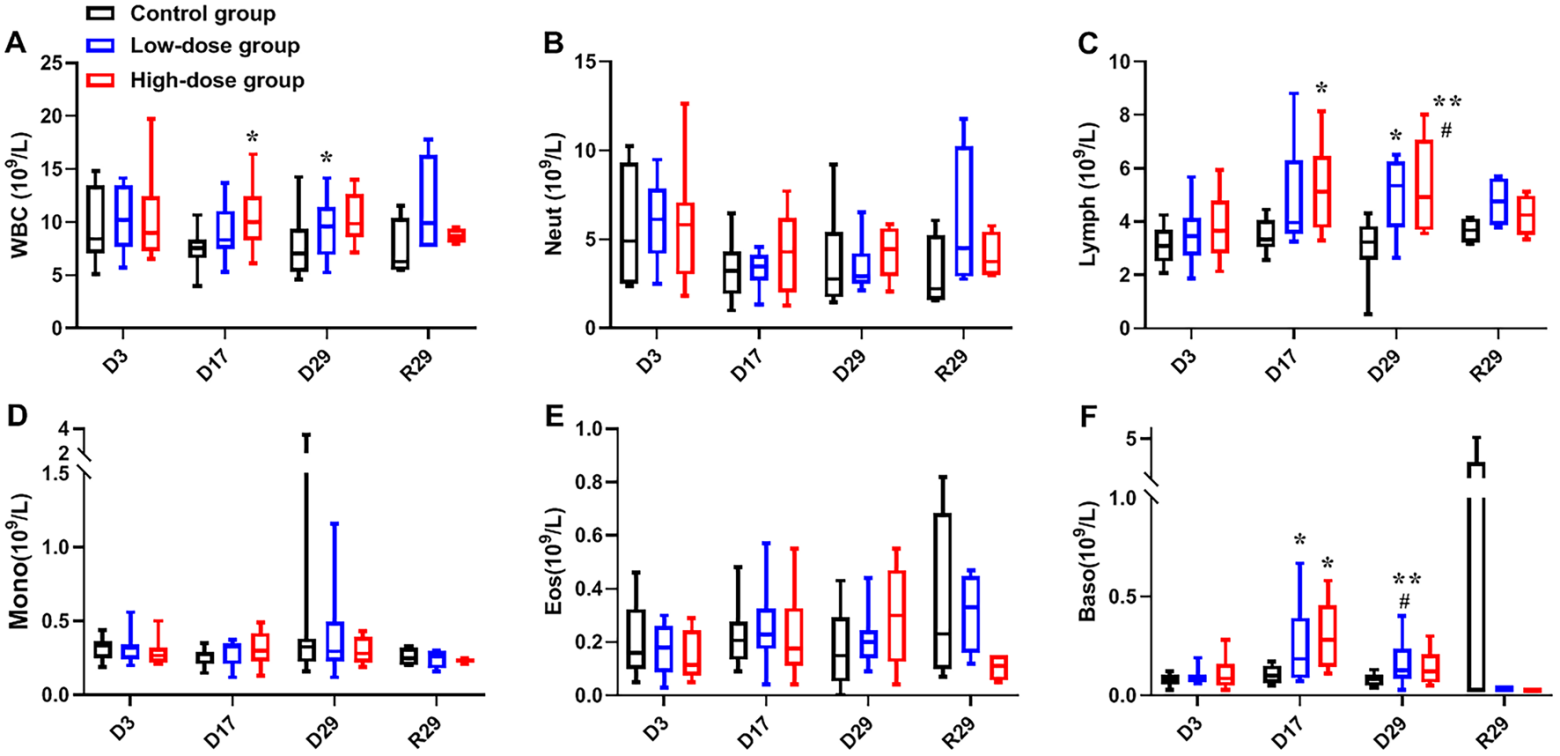
Changes on leukocyte and related classification after repeated administration of hUC-MSCs. Averaged WBC (**A**), Neut (**B**), Lymph (**C**), Mono (**D**), Eos (**E**), and Baso (**F**) count during the study are shown. (n = 10/group during dosing period; n = 4/group during recovery period). Data are presented as means ± SD. */#*P* < 0.05, **P < 0.01. #represents significant difference compared to Control group and High-dose group; *represents significant difference compared to Control group and Low-dose group.

**Figure 7:**
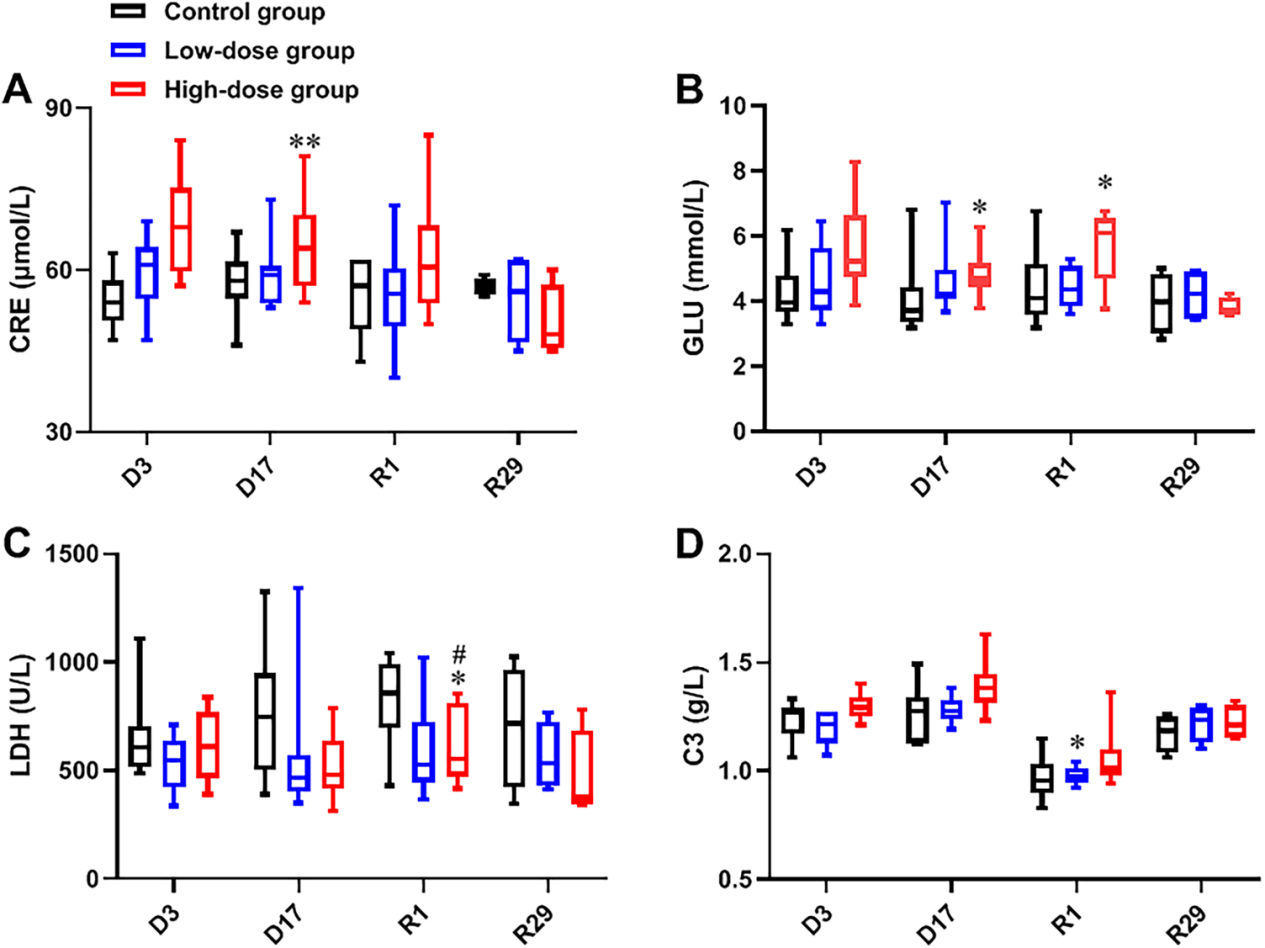
Changes on serum biochemical indexes after repeated administration of hUC-MSCs. Averaged levels of CRE (**A**), GLU (**B**), LDH (**C**), and C3 (**D**) during the study are shown. (n = 10/group during dosing period; n = 4/group during recovery period). Data are presented as means ± SD. */#*P* < 0.05, **P < 0.01. #represents significant difference compared to Control group and High-dose group; *represents significant difference compared to Control group and Low-dose group.

Hematology and Serum Biochemistry data of animals administered hUC-MSCs were demonstrated in Figures 6 and 7, respectively. On D17, the levels of WBC, Lymph, Baso in the High-dose group were significantly higher than that in the Control group, Baso in the Low-dose group was significantly higher than that in the Control group. On R1, Lymph in both Low-dose group and High-dose group were significantly higher than that in the Control group, and the increase of Lymph was not observed by the end of recovery period.

CRE and GLU in the High-dose group were significantly higher than that in the Control group on D3. C3 in the High-dose group was significantly higher than that in the Control group on D17. LDH in both Low-dose group and High-dose group were significantly higher than that in the Control group on R1. GLU in the High-dose group were significantly higher than that in the Control group on R1.

No change on other hematological, serum biochemistry indexes as well as the uinalysis indexes of animals administered hUC-MSCs was found.

### Immunotoxicity and immunogenicity

**Figure 8:**
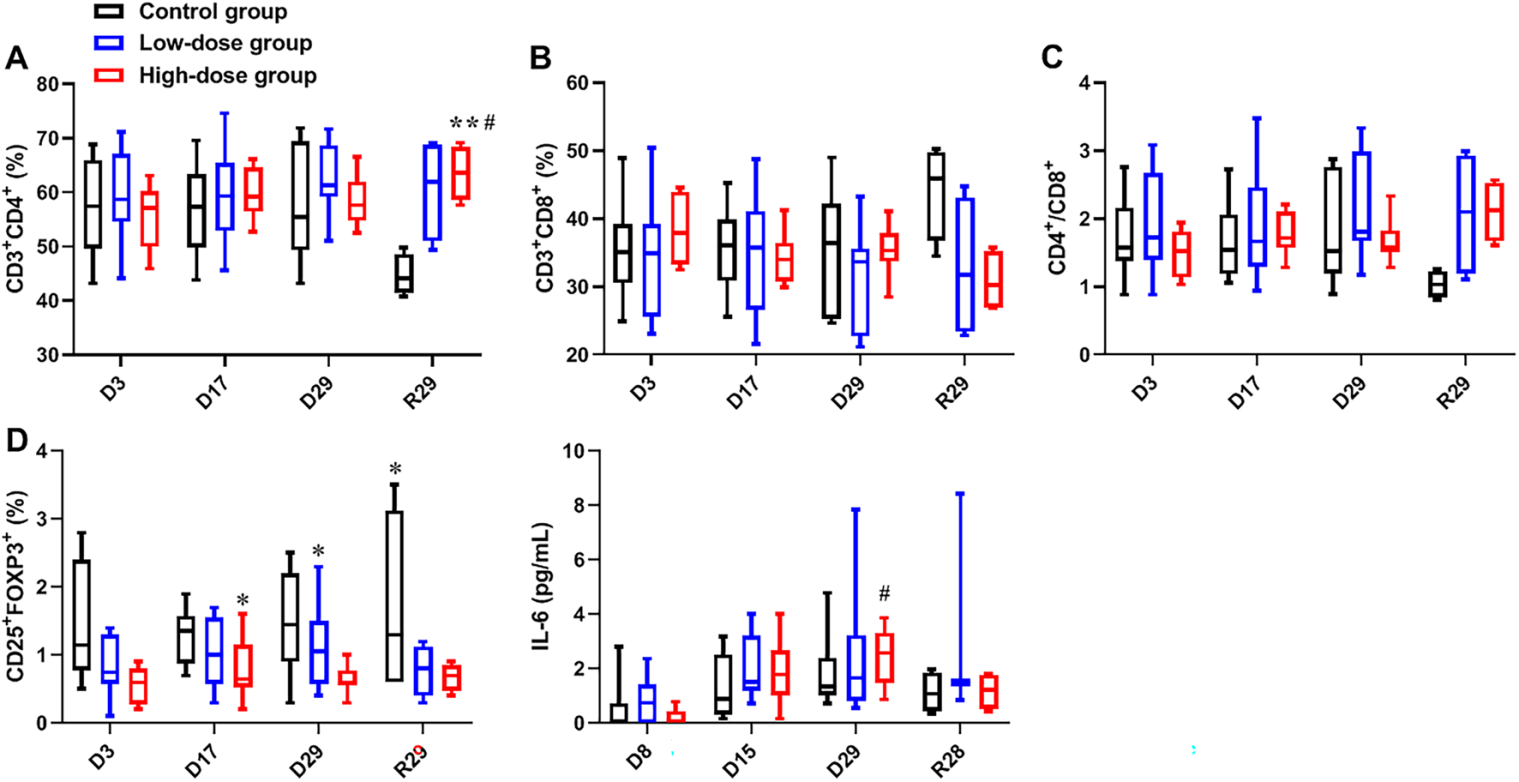
Changes on T lymphocytecount and IL-6 levels after repeated administration of hUC-MSCs. The percentage of CD3^+^CD4^+^ T lymphocytes (**A**), CD3^+^CD8^+^ T lymphocytes (**B**), CD4/CD8 (**C**) ratios and the percentage of CD4 + CD25 + Foxp3 + regulatory T lymphocytes (**D**) in peripheral blood, and levels of IL-6 in serum (**E**). Data are presented as means ± SD (n = 10/group in dosing period; n = 4/group in recovery period). */#*P* < 0.05, **P < 0.01. #represents significant difference compared to Control group and High-dose group; *represents significant difference compared to Control group and Low-dose group.

The changes on the T lymphocytes count were summarized in Fig. 8. CD3^+^/CD4^+^ T lymphocyte in both the Low-dose group and High-dose group were significantly higher than that in the Control group on R29. This change had no biological significance, as it is mainly attributed to the decrease in the Control group. On D3, D17, and D29, the count of Treg cell (CD25^+^Foxp3^+^) in the High-dose group was significantly lower than that in the Control group. The cells used in this study were human-derived stem cells. The above changes suggested that the monkeys developed xenogeneic immune rejection.

IL-6 in the High-dose group was significantly higher than that in the Control group on D29 (Fig. 8). Combined with the overall data, this difference might due to the individual differences of animals. No other change on the cytokine profile was found.

Anti-hUC-MSCs antibodies were failed to be detected in any monkey on D3, D8 and D15. By end of the administration (D29), the antibodies were found in 9 animals in both Low-dose group and High-dose group. Further, by end of the recovery period (R29), the antibodies were detected all the animals in both Low-dose group and High-dose group. A certain time-effect relationship of the generation of antibodies was observed (Table 1).

**Table 1:** Serum anti-hUC-MSCs binding antibodies detection results in monkey.

Group	Number of positive animal/number total animal			
	D8	D15	D29	R29
Control group	0/10	0/10	0/10	0/4
Low-dose group	0/10	0/10	9/10	4/4
High-dose group	0/10	0/10	9/10	4/4

### Histopathology

**Figure 9:**
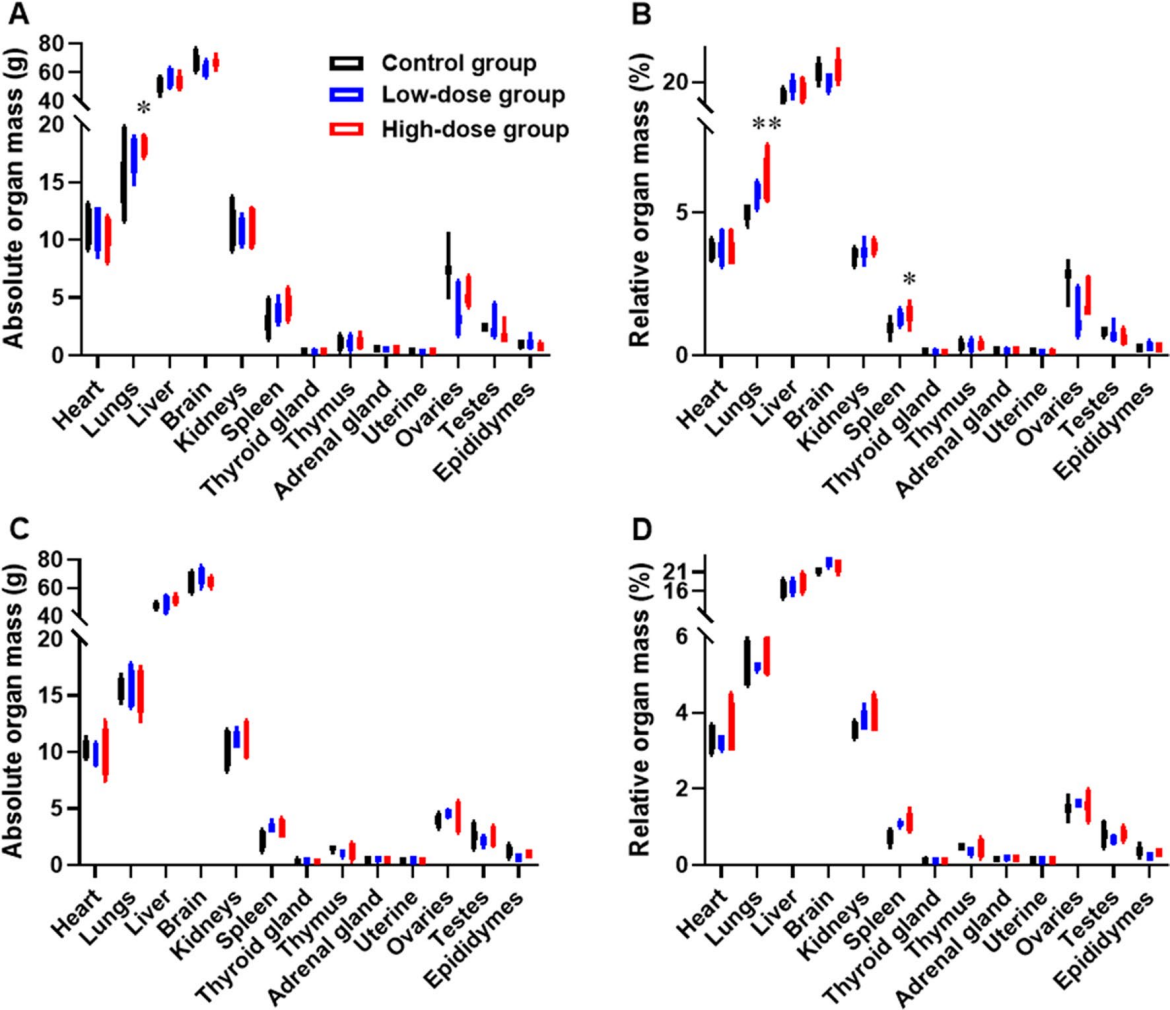
Organ mass measured by the end of dosing period and recovery period. (**A**) Absolute organ mass by end of dosing period. (**B**) Relative organ mass by end of dosing period. (**C**) Absolute organ mass by end of recovery period. (**D**) Relative organ mass by end of recovery period. Data are presented as means ± SD (n = 6 monkeys/group in treatment period; n = 4/group for recovery period). *P < 0.05, **P < 0.01. *represents significant difference compared to Control group and Low-dose group.

By the end of the dosing perid, absolute and relative liver mass in the High-dose group was increased compared to Control group, and relative spleen mass in the High-dose group was increased compared to Control group (Fig. 9). These lesions were recovered by the end of recovery period. Although histopathological examination showed that no changes associated with the administration of hUC-MSCs were observed in the lungs and spleens, the changes of the absolute and relative lungs masses in the High-dose group may relate to the bio-distribution of hUC-MSCs. The changes of relative spleen mass may relate to the immune rejection of hUC-MSCs on xenogeneic animals.

**Figure 10:**
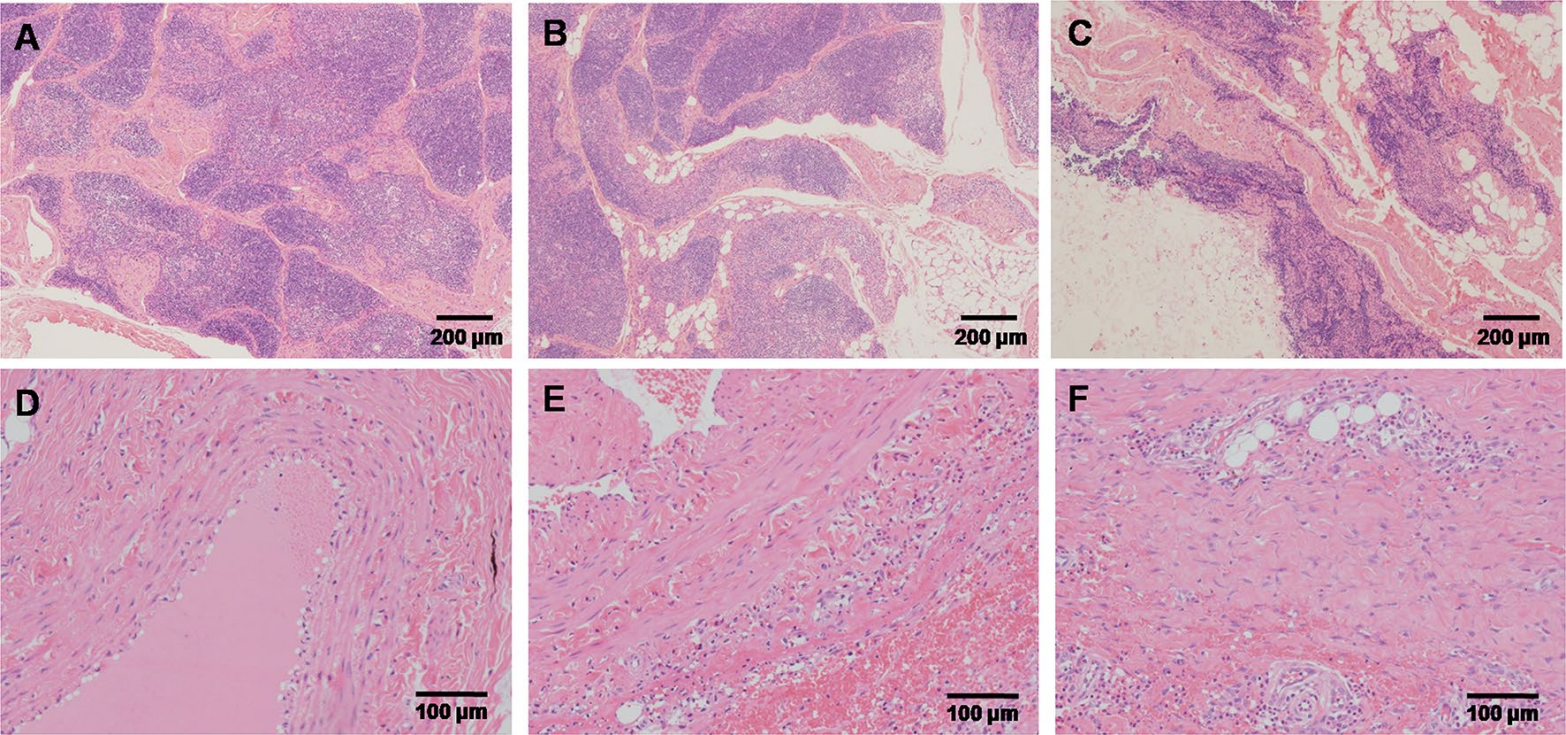
Histopatholoic changes in thymus and injection sites. (**A**,**B**) A mild to moderate decrease in lymphocytes in the thymic cortex in high dose group (4×). (**C**) A a mild decrease in lymphocytes in the thymic cortex in vehicle control group (4×). (**D**) Normal injection site (10×). (**E**) A mixed but predominantly neutrophilic inflammatory cell infiltrate appeared in the dermis in dermal or dermal–subcutaneous site in vehicle control group (10×). (**F**) A mixed but predominantly neutrophilic inflammatory cell infiltrate appeared in the dermis in dermal or dermal–subcutaneous site in high dose group (10×).

In High-dose group, the thymus gland volume was found reduced in two animals. Histopathological examination revealed a mild to moderate decrease in lymphocytes in the thymic cortex (Fig. 10A,B). Meanwhile, a mild decrease in lymphocytes in the thymic cortex in one monkey in Control group (Fig. 10C). There was very mild increase of macrophage in the spleen white pulp in some animals of High-dose group. There was also very mild increase macrophage in the spleen white pulp in some animals in Control group. A mixed but predominantly neutrophilic inflammatory cell infiltrate appeared in the dermisin dermal or dermal–subcutaneous site in each group (Fig. 10D–F).

## Discussion

Stem cells are undifferentiated cells possessing different degrees of self-renewal and multidirectional differentiation potential. Stem cells can proliferate in an undifferentiated state in vitro and differentiate into specific functional cells^22^. Currently, stem cells have shown promise in the treatment of malignant tumors, hematological diseases, neurological diseases, autoimmune diseases, metabolic diseases, cardiac diseases, ophthalmic diseases and skin diseases^23–26^. However, the toxicity risks of stem cells have received wide attention, and sufficient preclinical safety research is a necessity for providing safety data and ensuring the safety of human use in clinical trials.

Currently, there are few reports on the distribution and migration of hUC-MSCs in vivo. To understand the distribution characteristics of hUC-MSCs in vivo after cell transplantation, we used the fluorescent dye DiR to label hUC-MSCs before infusioninto monkeys. Flow cytometry method was used to detect the expression of DIR-labeled hUC-MSCs in the peripheral blood of monkey. DiR is a fluorescent dye with fluorescence located in the near-infrared region and could insert two 18-carbon chains into the cell membrane to form a stable label^27–30^. Despite that the detection period for dye labeling tracer is relatively short, it is a convenient, non-invasion method, and allows a real-time dynamic imaging. As demonstrated in our previous study (data not shown), both in vivo imaging and immunofluorescence method were simultaneously applied to detect the distribution and metabolism of UC-MSC in mice. The DiR could provide sensitive and reliable information for at least 2 weeks. Data obtained from the two methods were mutually verified, and together made the data more convincing. The DiR could stably label MSCs without an obvious effect on the biological characteristics of the cells, including morphology, viability, cell cycle and cell proliferation. We achieved dynamic detection of hUC-MSCs in the blood of monkey via flow cytometry. Our data showed that hUC-MSCs reached a small peak 0.083 h after dosing, and the number of cells in the blood gradually reached the peak after 1 day. These results indicted that after intravenous injection, some hUC-MSCs were distributed throughout the body within 0.5 h, while most of the cells accumulated in the lungs along with the systemic blood circulation, and were subsequently released into the blood. The number of cells in the blood slowly diminished after 2 days of dosing, due to the metabolic apoptosis.

Subsequent to 5 repeated administrations of hUC-MSCs, only a few animals in the High-dose group showed low copies of hUC-MSCs in peripheral blood at 0.5 h to 1 h and 0.5 h to 3 h after the first and the 5th administration, respectively. At the other time points, stem cell gene copy was not detected in peripheral blood and tissues. There was no significant increase in the gene copy number in the peripheral blood of patients by the end of treatment compared with the mesenchymal stem cell.

The proposed clinical administration frequency of hUC-MSCs is once per week and may be repeatedly administered for four times. In this study, monkeys as non-human primates were used, and hUC-MSCs were administered intravenously once a week for 5 consecutive months to observe the toxicity and severity that might be caused by hUC-MSCs. Toxicity studies in animals is required to simulate the clinical dosing schedule to provide information for the design of clinical trials, including the optimal clinical dose, dosing period, route and frequency. Factors of great importance include the justifications of the animal, dose, and dosing schedule. Cynomolgus monkeys, as non-human primates, are genetically similar to humans, and abundant background data under physiological conditions have been accumulated. In terms of dose selection, the proposed clinical dose of hUC-MSCs is 1 × 10^6^ cells/kg, which is equivalent to 2.6 × 10^6^ cells/kg in monkey. Hence, 3 × 10^6^ cells/kg and 2 × 10^7^ cells/kg (approximately 8 times of the clinical dose) were used as the low dose and high dose respectively in this study. As previously reported, the MSCs were repeatedly administered to monkeys for 4 weeks (once per week) or 16 weeks (once per 2 weeks) at up to 2 × 10^7^ cells/kg, and no severe toxicity was observed^31–34^. As demonstrated in our study, the monkeys were tolerated at both doses well. Intravenous injection has been proposed to be the best route for administrating hESC-MSCs to patients, which we also used here in the monkeys to avoid an alteration in efficacy caused by a different administration route. According to the number of times of clinical application, the frequency of drug administration was set as one time per week, and the drug was given at 0,1,2,3,4 weeks respectively. The recovery period is set at 4 weeks.

The administration of MSCs had no effect on clinical symptoms. Redness at the injection site was observed in some animals among groups, which could be recovered at recovery period. Histopathological examination confirmed that the above-mentioned redness at the injection site was related to mechanical stimulations of intravenous administration, and was not correlated with the direct effect of hUC-MSCs. hUC-MSCs reduced the number of Treg cells.This might related to the immunologic reactions of the allograft rejection. There are also no MSCs-related changes in body weight, body temperature, blood pressure, hematology, serum biochemistry, urinary analysis, and cytokines among groups. On D28 and R29, animals detected anti-hUC-MSCs antibodies in Low and High-dose groups. The timepoints for antibody production was in accordance with the general regularity that the body was induced to produce IgG antibodies upon antigen stimulation. Previous study has demonstrated that hUC-MSCs have certain immunogenicity in cynomolgus monkeys. By the end of the dosing period, absolute and relative liver masses were increased in the High-dose group compared to Control group, and relative spleen mass was increased in the High-dose group compared to Control group, which was recovered by the end of recovery period. Studies have reported that mesenchymal stem cell was distributed in varying proportions in the lungs and liver after transplantation^35–37^. Although no MSCs-associated histopathological lesions were found on lungs and spleen in High-dose group, the increased mass of the lungs and spleen might be related to the administration of hUC-MSCs. This effect on lungs might relate to the bio-distribution of hUC-MSCs. The increased mass of the spleen might relate to the xenogeneic immune rejection of hUC-MSCs.

## Conclusion

In conclusion, hUC-MSCs at low and high doses had no significant effect on the clinical symptoms, injection site, body mass, body temperature, ECG, blood pressure, serum biochemistry, urinary biochemistry examination and cytokine levels of the animals. However, abnormal changes associates with the bio-distribution and xenogeneic immune rejection of hUC-MSCs were observed, including the decreased of Treg cells, mass change of lungs and spleen, and the production of anti-hUC-MSCs antibody, and these were all recovered by 4 weeks after the 5th administration. The above changes may be related to the distribution of hUC-MSCs in the lungs and the xenogeneic immune rejection of human-derived stem cells in mesenchymal stem cell of monkeys. Nevertheless, this study indicated that much attention should be paid during the clinical use especially in immune rejection of hUC-MSCs. These data revealed the distribution and toxicological profile of hUC-MSCs and supported investigational new drugapplicationfor further clinical trials. This study provided valuable preclinical data for the safety of hESC-MSCs in the treatment of major refractory diseases, such as endometrial aplasia, and supports the marketing of hUC-MSCs in the form of new drugs.

### Originality statement

All the figures and table in this article have not been published previously, and not under consideration for publication elsewhere, in whole or in part.

## Data Availability

The data used to support the findings of this study are included within the article.
